# Does Imitation Facilitate Word Recognition in a Non-Native Regional Accent?

**DOI:** 10.3389/fpsyg.2012.00480

**Published:** 2012-11-12

**Authors:** Noël Nguyen, Sophie Dufour, Angèle Brunellière

**Affiliations:** ^1^UMR 7309, Laboratoire Parole et Langage, CNRS, Aix-Marseille UniversitéAix-en-Provence, France; ^2^Laboratoire URECA, Université de Lille IIIVilleneuve d’Ascq, France

**Keywords:** speech, imitation, spoken word recognition, French, regional variety

## Abstract

We asked to what extent phonetic convergence across speakers may facilitate later word recognition. Northern-French participants showed both a clear phonetic convergence effect toward Southern French in a word repetition task, and a bias toward the phonemic system of their own variety in the recognition of single words. Perceptual adaptation to a non-native accent may be difficult when the native accent has a phonemic contrast that is associated with a single phonemic category in the non-native accent. Convergence toward a speaker of a non-native accent in production may not prevent each speaker’s native variety to prevail in word identification. Imitation has been found in previous studies to contribute to predicting upcoming words in sentences in adverse listening conditions, but may play a more limited role in the recognition of single words.

## 1 Introduction

In current research on spoken communication, a major challenge is to better understand how spoken language is produced and perceived by humans in the context of what is regarded as its primary site of occurrence, i.e., social interaction. Whereas earlier research has traditionally focused on laboratory speech, produced by single individuals, more recent work has provided evidence strongly suggesting that the way in which language is used in everyday conversational exchanges, has a direct impact on how it is cognitively represented. Usage-based models of language (Barlow and Kemmer, 2000; Couper-Kuhlen and Ford, 2004; Bybee, 2006) very much emphasize the social dimension of language, and contend that the cognitive representations that are brought into play in the production and processing of spoken language do not entirely preexist to the interactions that may take place between talkers, but are rather subject to a co-construction process in which both interactants are engaged.

In recent years, a growing number of studies have centered on speech patterns in conversational interaction (e.g., Couper-Kuhlen and Ford, 2004). A major issue of interest in these studies is the tendency shown by participants in a conversation to imitate each other. Convergence effects have been shown to be systematic and recurrent, and manifest themselves under many different forms, which include posture (e.g., Shockley et al., 2003), head movements and facial expressions (e.g., Estow et al., 2007; Sato and Yoshikawa, 2007) and, as regards speech, vocal intensity (Natale, 1975), pitch curve (Gregory et al., 1993; Bosshardt et al., 1997), and rate of speech (Giles et al., 1991). In a recent, seminal work, Pardo (2006) has shown that perceived similarity in pronunciation between talkers increases over the course of the interaction and persists beyond its conclusion. These phenomena may facilitate conversational exchange by contributing to setting a common ground between speakers (Giles et al., 1991). They may have the same effect as so-called alignment mechanisms, assumed to apply to linguistic representations at different levels between partners, in order for these partners to have a better joint understanding of what they are talking about (Garrod and Pickering, 2004).

While imitation occurs, by definition, within a social interaction, it has consequences for language that extend much beyond the temporal limits of that interaction. According to Studdert-Kennedy (2002) and Goldstein (2003), it plays a central role in the acquisition of phonology, among the many aspects of language development. Imitation may also form one of the key mechanisms that underlie the emergence and evolution of human languages (de Boer, 2000). In addition, imitation in humans embraces a domain that is of course much wider than that of language itself. In Piaget’s theory, it is associated with a key stage of child development, between the sensorimotor stage and the formation of the first mental images. On the basis of infants’ observed capacity to mimic facial expressions from the very first days after birth, Meltzoff and Moore (1997) have hypothesized that imitation is central to the development of self in relationship with others. At the brain level, an increasingly large number of studies today concentrate on the links that may exist between imitation and mirror neurons, whose recent discovery has raised important issues about the role that these neurons may fulfill in many different domains, from sensorimotor integration to the understanding of others’ behavior (Rizzolatti and Craighero, 2007).

In psycholinguistic research, these effects have often been used as a probe for exploring the plasticity of the representations for words in the mental lexicon under exposure to another speaker’s voice. As Goldinger (2000) put it, “the imitation data verify that the contents of memory can be reflected in the sound of a person’s voice.” Imitation effects were found to be greater for word tokens that had been previously heard more often by the speaker, in an immediate shadowing task, a phenomenon that was attributed by Goldinger (1998) to the strengthening of the memory traces for the more often presented word tokens compared with the less often presented ones. Perceived phonetic convergence across speakers was also shown to be greater for low-frequency words than high-frequency words (Goldinger, 1998, 2000), and this has been interpreted as providing support for the existence of an episodic-memory component in word production and perception.

Phonetic convergence effects between speakers have been explored in both interactive (e.g., Pardo, 2006) and non-interactive, laboratory (e.g., Goldinger, 1998; Delvaux and Soquet, 2007) settings. Studies conducted in a non-interactive laboratory setting suggest that phonetic convergence toward another speaker is a highly automatized process that may be triggered upon hearing the other speaker’s voice, outside the domain of a conversational exchange. It also demonstrates that a representation of the other speaker’s individual and social identity is automatically formed and brought into play as the speaker’s voice is perceived and processed by the listener (e.g., Hay et al., 2006a,b). This is, in our view, powerful evidence for the role of phonetic convergence in speech production and perception, since convergence mechanisms occurring in a non-interactive setting are likely to be used by speakers to a yet larger extent in an interactive setting.

In this study, we took the non-interactive laboratory approach, and asked to what extent phonetic convergence across speakers may facilitate spoken word recognition. It may be assumed that convergence contributes to making each speaker more attuned to the phonetic characteristics of words produced by the other speaker, via a perception-action resonance phenomenon. In some theoretical frameworks (e.g., Pickering and Garrod, 2007), imitating an individual’s actions makes it easier to predict what that individual will do next, particularly in the case of ambiguous or distorted input. In a recent behavioral study, Adank et al. (2010) showed that imitation of a novel accent improves language comprehension in adverse listening conditions. They exposed Dutch-speaking participants to a novel accent under different conditions during a training phase, and assessed comprehension of the accent before and after training by measuring the signal-to-noise ratio at which listeners can repeat 50% of the key words in a sentence. The novel accent was a variant of Dutch that switched particular vowels in words such that the accent was unfamiliar to the native Dutch participants. The results showed that accented speech comprehension was improved only for participants who had imitated the speaker’s accent. When participants had to listen to the accented sentences, or to listen and transcribe them, or to listen and repeat them in the participant’s own accent, an improvement of comprehension was not observed. Together, these results suggested that language comprehension was indeed improved by vocal imitation, as opposed to other factors such as speaking out aloud or paying attention to the phonetic/phonological properties of the accented speech.

As in Adank et al. (2010), we examined the potential impact of imitation on the understanding of an unfamiliar accent. Unlike Adank and colleagues, however, we focused on the role that imitation may have in the identification of single words, as opposed to words in sentences. In addition, whereas Adank and colleagues used a non-existing accent that they created for the purpose of their study, we endeavored to determine how imitation may improve spoken word comprehension in an existing non-native variety of the listener’s native language. In our experiment, native speakers of Northern French were exposed to words produced by a native speaker of Southern French.

Well-established differences in the phonemic inventories associated with Northern French and Southern French include the /o/-/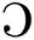/ contrast in word-final closed syllables that exists in Northern French but not in Southern French, which only has the open-mid variant in that position. In what we will refer to as *closed words* (CL-words), the vowel is realized as [o] in Northern French (e.g., *rose* [
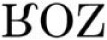
]) and as [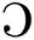] in Southern French ([
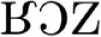
]), whereas in *open words* (OP-words), the vowel is realized as [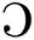] in both Northern French and Southern French (e.g., *robe* [
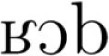
] “dress”; Durand, 1990; Durand and Lyche, 2004). We raised two questions. The first question was whether Southern-French productions of CL-words would be more difficult to recognize by Northern-French listeners than Southern-French productions of OP-words. Assuming that this is indeed the case, we further asked whether repeating CL-words as produced by a Southern-French speaker would later make it easier for Northern-French speakers to recognize these words.

To determine whether Southern-French CL-words would be more difficult to process than Southern-French OP-words, we presented both CL- and OP-words to a first group of Northern-French speakers in a lexical-decision task. To establish whether overt imitation has an impact on later recognition, we asked a second group of Northern-French speakers to perform a repetition task, which entailed repeating CL- and OP-words previously recorded by a Southern-French speaker, then the same lexical-decision task as for the first group. A third group of Northern-French participants did a semantic-categorization task, which entailed the presentation of the same acoustic tokens but no overt speech production, then the lexical-decision task. This group was used to disentangle the potential effect of simple exposure to the speaker’s speech from the one that overt imitation may show, on subsequent performance in the lexical-decision task. The first group will be referred to as the control group, the second one as the repetition group, and the third one as the categorization group. Assuming that overt imitation does facilitate later recognition, we predicted that the difficulty in processing CL-words would tend to decrease in the repetition group compared with both the control and the categorization groups. In a preliminary stage, the three groups of participants performed a reading-aloud task that allowed us to ensure that they all produced the /o/-/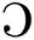/ contrast in word-final closed syllables. At the time of the study, all Northern-French speakers were living as students or young scholars in Provence, where Southern French is the dominant accent.

## 2 Materials and Methods

### 2.1 Participants

Three groups of fourteen (eleven women, three men) Northern-French speakers took part in the experiment. They were selected among undergraduate students and young scholars at the University of Aix-Marseille and reported no hearing or speech disorders. The mean age was 28.9 years for the control group, 25.1 years for the repetition group, and 26.5 years for the categorization group. Note that while the participants were all born and raised in the northern part of France, they were regularly exposed to Southern French as Aix-Marseille students and scholars.

### 2.2 Material

#### 2.2.1 Reading-aloud task

For the reading-aloud task, the material was made up of eight CVC minimal pairs each formed by an OP-word (e.g., *pomme*/
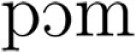
/ “apple”) and a CL-word (*paume* /*pom/* “palm”). Each word was embedded in a carrier sentence that differed across words. 64 filler sentences were also used.

#### 2.2.2 Repetition and semantic-categorization tasks

For the repetition and semantic-categorization tasks, we used 20 CL-words and 20 OP-words (see list in the Appendix). We selected these words in the VOCOLEX French lexical database (Dufour et al., 2002) so that they were matched with respect to lexical frequency and number of phonological neighbors. The uniqueness point in these 40 words fell on average after the last phoneme. None of the CL-words constituted a minimal pair with an existing OP-word in Northern French. This means that the Southern-French realizations of CL-words were bound to be perceived as little familiar CL-word forms, as opposed to OP-words, by the Northern-French participants. To divert the participants’ attention away from the /o/-/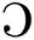/ constrast, we further selected 160 CVC filler words that contained neither of the two vowels. Values of the selection parameters for CL- and OP-words are given in Table 1.

**Table 1:** **Values of the selection parameters for CL-words and OP-words**.

	CL-words	OP-words
Number	20	20
Nb of phonemes	3	3
Mean position of uniqueness point in nb of phonemes	3.75	4
Mean lexical frequency (over one million)	54	54
Mean nb of phonological neighbors	26	28

#### 2.2.3 Lexical-decision task

For the lexical-decision task, we used the same 20 CL-words and 20 OP-words as in the repetition and semantic-categorization tasks, together with 160 CVC filler words that contained neither of the two /o/-/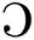/ vowels. The fillers were different from those in the repetition and semantic-categorization tasks, in order to minimize the number of words that were common to either the repetition or semantic-categorization task and the lexical-decision task.

For the needs of the lexical-decision task, we also created 200 CVC non-words by changing the last phoneme in real words, in order to constrain the participants to listen to stimuli up to the end prior to giving their response (see Vitevich, 2007, for the same procedure). Twenty of these non-words were derived from existing CL-words, and another twenty were derived from existing OP-words.

#### 2.2.4 Recordings

All CVC words and non-words were recorded twice by a native speaker of Southern French (the first author, see Nguyen and Fagyal, 2008, for detail on his accent), in the anechoic chamber of the Laboratoire Parole et Langage, using high-quality digital recording equipment at a sampling rate of 44100 Hz. We measured the acoustic duration of both repetitions for each item and selected either the first or second repetition so that, on average, differences in duration across CL-words and OP-words were minimized [*F*(1, 38) = 0.22, *p* > 0.2].

## 3 Experimental Procedure

The experiment took place in the anechoic chamber of the Laboratoire Parole et Langage. In the reading-aloud and repetition tasks, participants were recorded using the same equipment as for the Southern-French speaker. Auditory stimuli were played to the participants over headphones at a comfortable sound level.

During the repetition and semantic-categorization tasks, participants were presented with four blocks of 50 words, with a short break between each block and the following one. The critical trials were equally distributed across the blocks. The repetition group had to repeat the words as these had been produced by the Southern-French speaker. The categorization group was asked to press a button on a response box with their dominant hand as quickly as possible when the presented word belonged to a pre-specified semantic category (e.g., animal). Four semantic categories were used, one for each block. The ISI within each block was set to 2000 ms.

In the lexical-decision task, two blocks of 200 stimuli (100 words and 100 non-words) were presented to the participants, with an equal distribution across blocks of the critical trials. All participants were instructed to press one response box button with their dominant hand if the stimulus was a French word and another button with their non-dominant hand in the opposite case. Response times (RTs) were measured from the acoustic onset of the stimuli^1^. An interval of 1800 ms was allocated for the participant to respond, and there was a further 2000-ms interval between the participant’s response and the presentation of the following stimulus. Each phase began with ten practice trials.

## 4 Results

### 4.1 Acoustic data

A perceptual evaluation performed by a Northern-French expert phonetician showed that, as expected, all the participants clearly produced the /o/-/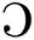/ contrast in the reading-aloud task.

Acoustic analyses were carried out for both the Southern-French speaker and the repetition group, to determine to what extent the repetition group converged toward the Southern-French speaker in the repetition task compared with the reading-aloud task. Acoustic recordings were segmented using Praat (Boersma, 2001). For each OP- and CL-word, we located the onset and offset of the vowel. *F*_1_ frequency was then automatically measured at the vowel’s acoustic midpoint using the Burg algorithm as implemented in Praat.

**Figure 1:**
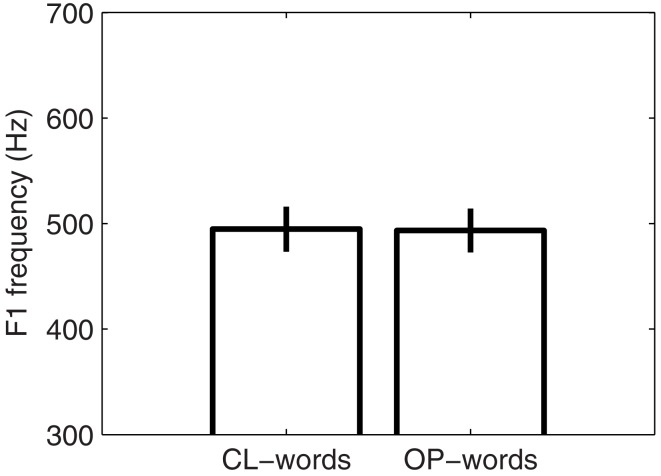
**Mean *F*_1_ frequencies and corresponding standard deviations for the vowel in the OP- and CL-words produced by the Southern-French speaker**.

In line with our characterization of Southern French, the Southern-French speaker displayed no significant difference in *F*_1_ frequency for the vowel in CL-words (mean *F*_1_ frequency: 494 Hz) compared with OP-words [mean *F*_1_ frequency: 493 Hz; *F*(1, 38) = 0.032, NS]. The mean *F*_1_ frequencies and corresponding standard deviations for the two word categories and for that speaker are shown in Figure 1.

**Figure 2:**
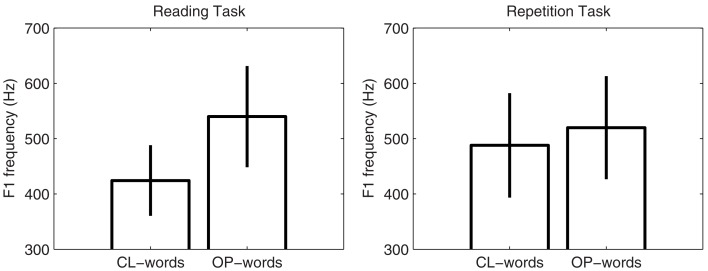
**Mean *F*_1_ frequencies and corresponding standard deviations for the vowel in CL- and OP-words for the repetition group in the reading-aloud and repetition tasks**.

For the Northern-French participants in the repetition group, we carried out repeated-measure two-way ANOVAs with word type (CL- vs. OP-words) and task (reading task vs. repetition task) as independent variables, both by subject and by item. *F*_1_ was found to be significantly higher in OP-words (mean frequency: 530 Hz) than in CL-words [mean frequency: 456 Hz; by subject: *F*(1, 13) = 111.85, *p* < 0.001; by item: *F*(1, 52) = 88.63, *p* < 0.001]. A significant word type × task interaction was also found [by subject: *F*(1, 13) = 46.23, *p* < 0.001; by item: *F*(1, 52) = 40.14, *p* < 0.001]. This interaction indicates that *F*_1_ frequency varied between CL- and OP-words to a lesser extent in the repetition task [by subject: *F*(1, 13) = 11.76, *p* < 0.01; by item: *F*(1, 38) = 22.65, *p* < 0.001] than in the reading-aloud task [by subject: *F*(1, 13) = 155.98, *p* < 0.001; by item: *F*(1, 14) = 89.01, *p* < 0.001]. In addition, the word type × task interaction shows that the Northern-French participants produced CL-words with a more open vowel [by subject: *F*(1, 13) = 8.97, *p* < 0.05; by item: *F*(1, 26) = 58.002, *p* < 0.001], in the repetition task (mean *F*_1_ frequency: 488 Hz) compared with the reading task (mean *F*_1_ frequency: 425 Hz), whereas OP-words were produced with a vowel that was open to the same degree [by subject: *F*(1, 13) = 2.549, NS; by item: *F*(1, 26) = 3.911, NS] in both the reading task (mean *F*_1_ frequency: 540 Hz) and the repetition task (mean *F*_1_ frequency: 520 Hz). Thus, in the repetition task, a clear convergence effect was shown by the Northern-French participants toward the Southern-French speaker. The mean *F*_1_ frequencies and corresponding standard deviations for the two word categories in each of the two tasks for these participants are shown in Figure 2.

### 4.2 Perceptual data

We first checked that the categorization group correctly processed the stimuli they were presented with in the semantic-categorization task. This was indeed the case as the participants reached an overall correct-response rate of 91%.

Perceptual data collected in the lexical-decision task were analyzed as follows. Three items that gave rise to an error rate of more than 40% were excluded from the analyses. For each participant and for each condition, reaction times (RTs) greater than 2 SDs above and below the participant’s average RT were also excluded (4.3% of the data). Incorrect responses were also removed from the RT analyses. The mean RTs and error rates in each condition are presented in Table 2. A log odds ratio transform was applied on error rates (Dixon, 2008; Jaeger, 2008). Analyses of variance (ANOVAs) by participants (*F1*) and by items (*F2*) were performed with group (control, repetition, categorization) and word type (CL-words, OP-words) as variables.

**Table 2:** **Mean RT values (in ms), corresponding standard deviations, and error rates (%), for the three groups of participants and for the two word categories in the lexical-decision task**.

		CL-words	OP-words
Control group	RTs	1079 (129)	1013 (135)
	Error rate	14.68	7.14
Repetition group	RTs	896 (86)	859 (80)
	Error rate	5.56	2.63
Categorization group	RTs	902 (102)	873 (124)
	Error rate	2.63	2.38

*Rts were measured from the words’ acoustic onsets. See text for details*.

#### 4.2.1 RTs analyses

The main effect of group was significant [*F*1(2, 39) = 11.43, *p* < 0.001; *F*2(2, 70) = 250.58, *p* < 0.001]. This effect was caused by the control group responding more slowly than both the repetition and the categorization groups. The main effect of word type was also significant [*F*1(1, 39) = 15.51, *p* < 0.001; *F*2(1, 35) = 4.91, *p* < 0.5]. On average, the CL-words were responded to more slowly than the OP-words. Although the RT difference between CL- and OP-words was reduced in the repetition and categorization groups compared with the control group, no interaction between groups and word type was found [*F*1(2, 39) = 0.98, *p* < 0.20; *F*2(2, 70) = 1.99, *p* < 0.14].

#### 4.2.2 Error analyses

The main effect of group was significant [*F*1(2, 39) = 14.99, *p* < 0.001; *F*2(2, 70) = 11.97, *p* < 0.001]. This effect was caused by the control group producing more errors than both the repetition and the categorization groups. The main effect of word type was significant by participants but failed to reach significance by items [*F*1(1, 39) = 8.29, *p* < 0.01; *F*2(1, 35) = 2.35, *p* < 0.13]. No interaction was found (*p*s > 0.10).

## 5 Discussion and Conclusion

Our acoustic data revealed that Northern-French participants clearly tended to converge toward the Southern-French speaker in the realization of the /o/-/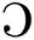/ contrast, in the repetition task compared with the reading task. We found a significant tendency for Northern-French participants to produce CL-words with a more open vowel in the repetition relative to the reading task, whereas OP-words were produced with a vowel that was open to the same degree in both tasks. However, our perceptual data showed that all Northern-French participants were slower and less accurate in processing Southern-French forms of CL-words than OP-words, whose pronunciation is similar in both Southern and Northern French. The repetition group showed no significant advantage over the categorization and control groups in response speed or accuracy for CL-words compared with OP-words. Note that the difference in RT between the two word types was in fact greater for the repetition group than for the semantic-categorization group, although this trend did not reach statistical significance. Thus, overt phonetic imitation did not appear to have a measurable impact on later word recognition in the present experiment.

Northern-French participants therefore showed both a tendency to converge toward Southern French in the word repetition task, and a bias toward the phonemic system of their own variety in word recognition. This may be consistent with the view that phonetic convergence is primarily an interactional device, used for reinforcing a sense of shared identity between speakers. Adaptation to the other speaker in production may not prevent each speaker’s native variety to prevail in word identification. Our data are also consistent with the view that there may be a decoupling between changes in production and changes in perception as the listener is exposed to different sources of phonetic variation (Kraljic et al., 2008).

The greater difficulty in processing non-native word forms may be accounted for by a frequency-based model of word processing and recognition, such as the one developed by Connine et al. (2008). On that account, both the native, more familiar (e.g., [
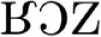
]) and the non-native, less familiar ([
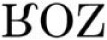
]) forms for CL-words would be stored in the listener’s mental lexicon, with a greater weight associated with the more familiar form. This would allow the more familiar form to be recognized more rapidly and with greater accuracy than the less familiar one.

Previous work such as the one by Maye et al. (2008) suggests that perceptual adaptation to a novel accent can occur following a short period of exposure to that accent, and in the absence of overt repetition and imitation. Because our participants were regularly exposed to Southern French in their daily life, it may well be the case that they had all undergone perceptual adaptation to Southern French, to a certain degree at least, at the time of the experiment. Importantly, however, the participants’ poorer performance in the recognition of less familiar CL-word forms compared with more familiar OP-word forms indicated that such a perceptual adaptation to Southern French was in any case incomplete. Among the several differences that exist between Maye et al.’s (2008) experimental design and ours, one particularly important one relates to the fact that Maye and colleagues used a novel, artificial accent that they created by shifting down the entire set of front vowels of standard American English in the *F*_1_–*F*_2_ vowel space. As a result, whereas all front vowels were lowered, acoustic differences between vowels were maintained for most vowel pairs. Our own study centered on how speakers of Northern French, which contains a close-mid /o/ vs. open-mid /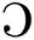/ phonemic contrast, would process words produced by a speaker of Southern French, which does not have that contrast. Thus, perceptual adaptation to an existing non-native accent may be difficult, particularly when the native accent has a phonemic contrast that is associated with a single phonemic category in the non-native accent.

In a recent study, Adank et al. (2010) found evidence that imitation of an unfamiliar accent improves spoken language comprehension in that accent. They created a non-existent accent of Dutch by asking a trained phonetician to systematically alter the pronunciation of all Dutch vowels embedded in sentences. As in the Maye et al. (2008) study, a one-to-one mapping was established between native phonemic categories and their phonetic realization in the novel accent for most vowels. In our study, by contrast, there was a many-to-one mapping between the Northern-French CL-word and OP-word forms and the Southern-French ones, as pointed out above. Whether this is a relevant factor in the role that imitation may play in the comprehension of non-native accents is a matter for future investigation. However, another major difference between Adank et al. (2010) and the present study is that we explored the potential impact that imitation may have on response speed in single word recognition, whereas Adank and colleagues focused on the role of imitation in the comprehension of sentences, and evaluated the listeners’ comprehension skills using a sentence intelligibility measure. According to Adank et al., it is indeed over the domain of the sentence that imitation would contribute to making an unfamiliar accent easier to understand. In agreement with Pickering and Garrod’s (2007) general hypothesis that imitation allows a listener to better predict what his/her conversational partner will say next, Adank et al. suggest that prior imitation of an unfamiliar accent helps the listener anticipate upcoming words in a sentence spoken in that accent. On that account, imitation may have a more limited contribution in the processing of words that have been or are being heard, as well as in the processing of single words. Yet another aspect of Adank et al.’s (2010) experiment is that the stimuli were presented with background noise, while this was not the case in the present work. As suggested by Adank et al. it may be possible that imitation facilitates spoken language comprehension in adverse listening conditions only.

To sum up, convergence toward a non-native accent was found to be fast and systematic in a repetition task. However, it did not prevent the speaker’s native accent from prevailing upon the non-native accent in spoken word processing. Imitation, whether overt or covert, has been found in previous studies to contribute to predicting upcoming words in sentences in adverse listening conditions, but may play a more limited role in single word recognition.

## Conflict of Interest Statement

The authors declare that the research was conducted in the absence of any commercial or financial relationships that could be construed as a potential conflict of interest.

## Footnotes

^1^Similar results were obtained when the RTs were measured from the acoustic offset of the stimuli.

## Appendix

List of the CL-words and OP-words used in the repetition, semantic-categorization, and lexical-decision tasks.

**Table:**

CL-words	OP-words
Baume	Choc
Cause	Corps
Chaude	Dot
Chaume	Gomme
Chauve	Loge
Cône	Moche
Dose	Mode
Faune	Noce
Fauve	Nonne
Gauche	Nord
Jaune	Note
Mauve	Phoque
Môme	Poche
Pause	Pop
Rôle	Pote
Rose	Robe
Sauce	Roche
Sauge	Tonne
Tôle	Vogue
Zone	Vote
